# The comfort paradox in emotional AI: how AI-mediated anxiety regulation may foster psychological dependence

**DOI:** 10.3389/fpsyg.2026.1723183

**Published:** 2026-06-19

**Authors:** Anish K. R., Jaya Cherian, Jeena Joseph, Jobin Jose

**Affiliations:** 1 Department of Social Work, Rajagiri College of Social Sciences, Kerala, India; 2 Department of Social Work, Vimala College Autonomous, Kerala, India; 3 Department of Computer Applications, Marian College Kuttikkanam Autonomous, Kerala, India; 4 Department of Library, Marian College Kuttikkanam Autonomous, Kerala, India

**Keywords:** anxiety, artificial empathy, comfort technology, digital dependence, emotional regulation, human–AI interaction

## Introduction

In contemporary digital wellness environments, emotional regulation technologies have become increasingly commercialized. Artificial intelligence now mediates how humans breathe, rest, focus, and even feel. From mindfulness apps that count calm min to voice companions offering nightly reassurance, AI has entered the most intimate domain of psychological life—the management of anxiety (Angelucci et al., 2024). These systems promise serenity through instant feedback and adaptive empathy. These systems provide affectively attuned feedback, detect markers of vocal or physiological stress, and generate personalized breathing or calming interventions based on user data (Rezk, 2023).

The systems under consideration include contemporary emotional and conversational AI tools used in digital wellness contexts, such as therapeutic chatbots that simulate empathic dialogue, mindfulness and mood-tracking applications that provide adaptive feedback, wearable-integrated stress dashboards that monitor physiological signals, and generative AI companions offering on-demand reassurance. Although these platforms vary in technological sophistication, they share a common structural feature: the automated delivery of personalized emotional support through real-time data processing and behavioral reinforcement mechanisms. The analysis concerns general adult users engaging with such systems for anxiety management. This article is presented as an opinion-based conceptual synthesis rather than as an empirical study, integrating established research in emotion regulation, reinforcement learning, neurobiology of reward processing, and human-AI interaction to introduce the comfort paradox as a framework for understanding how AI-mediated emotional soothing may shape anxiety regulation, psychological dependence, and resilience. The psychological and neurocognitive claims advanced are interpretive extensions grounded in prior empirical literature.

The argument advanced here is anchored in established empirical research on negative reinforcement, avoidance conditioning, and emotion regulation, and is extended cautiously to contemporary emotional AI systems such as therapeutic chatbots, adaptive mindfulness applications, and wearable-integrated stress platforms. The framework does not assume uniform effects across users. Its operation is likely moderated by developmental stage, baseline regulatory capacity, and sociocultural norms governing emotional expression and reassurance-seeking. By specifying these technological and contextual boundaries, the comfort paradox is positioned as a conditional mechanism rather than a generalized critique of AI-mediated support.

Yet beneath this experience of immediate subjective relief lies a psychological paradox. The more effortlessly AI reduces anxiety, the more it sustains the underlying dependency on reassurance. Repeated reliance on AI-mediated reassurance may reinforce avoidance-based coping patterns and increase dependence on external emotional regulation (Singh et al., 2024). This dynamic is conceptualized here as the comfort paradox—a psychological phenomenon in which digital comfort alleviates anxiety in the moment but weakens emotional resilience over time (Poenaru, 2024).

For clarity, the “comfort paradox” does not suggest that AI-mediated emotional support is inherently harmful. Rather, it refers to a specific psychological dynamic: when immediate, frictionless reassurance becomes the dominant mode of regulation, repeated reliance may reduce exposure to uncertainty and diminish opportunities for effortful coping. The paradox emerges not from the presence of comfort itself, but from patterns of habitual substitution of internal regulation with external automation. In this sense, the framework identifies a conditional risk mechanism rather than a deterministic outcome.

While the discussion draws on insights from neuroscience, affective computing, and human–AI interaction, the primary focus of this article remains psychological rather than technical. These interdisciplinary perspectives are used selectively to illuminate a single core concern: how AI-mediated soothing reshapes emotional regulation, dependence, and resilience. Rather than offering parallel analyses across domains, the article integrates these perspectives to deepen understanding of the psychological consequences of comfort-oriented AI.

This paper argues that although AI-mediated comfort may provide valuable emotional support, repeated reliance on such systems may reduce opportunities for internal emotional regulation and tolerance for uncertainty. The argument proceeds through six themes: the psychology of soothing, the paradox of dependence, algorithmic avoidance, the illusion of control, the loss of effortful emotional autonomy, and a concluding reflection on how to reclaim discomfort as a form of intelligence.

The comfort paradox is proposed as a conditional framework that reframes AI-mediated emotional support not merely as a regulatory tool, but as a reinforcement environment capable of reshaping long-term emotional habits. Extending established theories of avoidance and negative reinforcement into technologically mediated contexts, the framework highlights how scalable, frictionless, and continuously available reassurance may amplify reinforcement cycles. Its operation is not universal but shaped by design features such as immediacy of reassurance and the presence or absence of reflective prompts, as well as by user characteristics including developmental stage, baseline tolerance for uncertainty, and vulnerability to avoidance, and by patterns of usage ranging from occasional support to chronic reliance. The paradox is most likely to emerge under conditions of high-frequency use and minimal reflective friction, whereas systems that incorporate graduated autonomy, intentional delay, and time-limited support may attenuate reinforcement-based dependence. In this way, the framework specifies both a novel mechanism—algorithmically amplified negative reinforcement—and the boundary conditions under which it is expected to operate.

## The psychology of soothing

Anxiety, at its core, is an adaptive signal—a somatic preparation for uncertainty. Effective emotional regulation involves both soothing and integration: the first reduces arousal; the second helps the mind make meaning of threat. Integration requires the capacity to remain with discomfort long enough for insight to form. When relief arrives too early, learning halts (Thompson et al., 2019).

AI systems excel at the first half of this equation. Using affective computing techniques, these systems detect linguistic or physiological cues of distress and deliver brief, automated soothing interventions: affirmations, calming tones, or gentle prompts. These cues engage the body's parasympathetic response, lowering heart rate and cortisol, mimicking interpersonal co-regulation (Rebello, 2024). The relief feels authentic because the physiological pattern is genuine. But the underlying intentionality is absent; there is no consciousness sharing in one's pain (Jiang et al., 2022).

Psychologically, the transaction is instrumental rather than relational. The user receives comfort stripped of the mutual vulnerability that gives empathy its developmental power (Mantello et al., 2023). In this sense, instrumental soothing refers to a functional, goal-oriented process in which anxiety is reduced through automated responses designed to restore emotional equilibrium efficiently. Such regulation prioritizes outcome over interaction. In contrast, relational soothing arises from human encounters that involve mutual vulnerability, emotional presence, and shared meaning-making. While instrumental comfort can effectively reduce immediate distress, relational comfort supports emotional growth by allowing individuals to remain with discomfort long enough for reflection, learning, and resilience to emerge. Over time, such comfort conditions the user to seek regulation through external automation rather than internal reflection. Repeated reliance on automated reassurance may prioritize immediate relief over the development of internal coping capacity (Singh et al., 2024).

This shift mirrors trends observed in cognitive offloading. Just as people rely on digital reminders instead of memory, they may now rely on AI reassurance instead of affective endurance. Although immediate relief may be achieved, repeated external regulation may reduce opportunities for strengthening adaptive coping processes. When episodes of uncertainty are consistently met with automated reassurance mechanisms, individuals may become less practiced in initiating self-directed emotional regulation. Here, automated reassurance mechanisms refer to AI systems that provide immediate emotional support, such as conversational agents providing supportive language, mindfulness apps delivering calming prompts, or wearables triggering relaxation cues. While these tools can reduce anxiety in the moment, their repeated use may shift emotional regulation away from internal coping toward external automation.

## The paradox of dependence

At the behavioral level, the paradox operates through negative reinforcement. Reassurance, though comforting, is one of anxiety's most powerful reinforcers. In behavioral terms, it creates a negative-reinforcement loop: distress triggers reassurance-seeking; reassurance brings relief; relief reinforces the behavior (May et al., 2020). The more often the loop runs, the more tightly anxiety binds to the act of seeking comfort.

AI systems may intensify this mechanism by minimizing the effort required to obtain reassurance. Unlike a human friend or therapist, an app never sleeps or tires; reassurance is available on demand. Each episode of digital reassurance may resolve the anxiety cycle rapidly and with minimal effort from the user (Al-Refae et al., 2021). Users learn that discomfort is intolerable when not immediately soothed. As relief becomes easier to obtain, the threshold for anxiety lowers, producing the ironic outcome that more tools for calm generate more need for them (Barrios et al., 2022).

Empirical research on smartphone use supports this pattern: frequent reliance on calming or distraction apps correlates with increased baseline anxiety rather than reduction (Beaumont et al., 2025). The phenomenon is not dependence on devices *per se*, but on the emotional certainty they offer. A human conversation invites ambiguity; an algorithm offers precision. The predictability itself becomes addictive (Katz and Yovel, 2022).

This dynamic may contribute to increased reliance on external reassurance mechanisms for emotional regulation. The human nervous system, designed to oscillate between tension and release, now lives in an artificially flattened affective range—momentary calm punctuated by algorithmic reminders to stay calm. Theoretically, this paradox extends reinforcement-based models of anxiety by showing how AI-mediated reassurance functions as a scalable, frictionless form of negative reinforcement, shifting dependence from interpersonal reassurance to algorithmic regulation. This reframing highlights a novel mechanism through which technology reshapes emotional learning and anxiety maintenance.

Consider, for example, a user who turns to a conversational AI whenever anticipatory anxiety arises before a social interaction or during late-night distress. The system may immediately respond with empathic validation and guided calming prompts, offering breathing exercises and reassuring feedback about decreasing stress levels. While such interactions provide genuine short-term relief, they may do so without encouraging reflection, graded exposure, or exploration of the anxiety's source (Tsapekos et al., 2022). Over time, repeated episodes of rapid reassurance can strengthen the association between distress and external soothing, gradually narrowing tolerance for discomfort and reinforcing avoidance of internal processing (Liang et al., 2021). This pattern reflects the mechanism described by the comfort paradox: relief without integration.

## Algorithmic avoidance: when design teaches emotion

At the design level, the paradox becomes embedded in interaction architecture. Designers of emotional AI rarely intend harm. Their aim is retention: to keep users engaged through positive reinforcement. In this logic, calmness becomes a performance metric. Apps measure streaks of relaxation, offer badges for emotional stability, and gamify the absence of distress (Tejaswini Panse, 2025). Within some digital wellness systems, anxiety may increasingly be approached as a condition requiring immediate correction rather than reflective engagement.

In everyday use, these dynamics often manifest through recognizable interaction patterns: mood dashboards that visualize emotional “progress,” push notifications that offer unsolicited reassurance during detected stress spikes, streak-based reward systems reinforcing consecutive days of calm practice, or conversational prompts that immediately validate distress without encouraging reflective engagement. While designed to support wellbeing, such features illustrate how rapid reassurance and frictionless feedback may unintentionally reduce opportunities for uncertainty tolerance and effortful coping (Idrees et al., 2024; Torous et al., 2025).

From a learning perspective, this constitutes large-scale avoidance conditioning. Each time a system removes micro-frustration, it prevents exposure, the process by which fear responses naturally extinguish (Salil et al., 2025). In this context, avoidance conditioning does not simply refer to rapid soothing, but to a learned pattern in which individuals repeatedly bypass internal emotional processing by relying on immediate external regulation. When AI systems remove micro-frustrations too quickly, they reduce opportunities for exposure to uncertainty and distress—processes through which anxiety is typically integrated and diminished over time. As a result, users may come to prefer algorithmic relief over effortful, internal coping, reinforcing avoidance rather than emotional resolution. A generation raised with comfort algorithms may thus learn that negative feelings signify malfunction, not meaning. Instead of cultivating resilience, users may develop patterns in which elements of emotional regulation become externally mediated through technological systems.

Avoidance has moral and developmental costs. True empathy demands effort; it involves sitting with another's discomfort and risking one's own. AI-mediated empathy may provide emotional reassurance without the reciprocal vulnerability typically associated with human interaction. The algorithm predicts distress and responds before genuine connection or reflection can occur. Over time, this automation of empathy may recalibrate cultural expectations of care. People may come to equate feeling heard with being algorithmically mirrored. What disappears is not only the authenticity of care but its moral texture—the sense that compassion requires choice and labor (Mantello et al., 2023).

## The illusion of control

At the cognitive level, the paradox reshapes appraisal processes. Modern wellness technologies promise mastery over emotion. Dashboards monitor stress, sleep and “mood trends.” Thus, the signal is straightforward: by accumulating sufficient data, peace can be produced. However, emotional existence is not a factor to be maximized but a pattern to be comprehended.

The assumption that anxiety can be monitored and controlled entirely through metrics only strengthens what psychologists refer to as the control illusion-the misbelief of one's power to control inner states through external adjustments is exaggeration (Messina et al., 2023). Such illusion provides momentary certainty but erodes acceptance, the cornerstone of long-term regulation. When users see spikes in their anxiety graphs, they perceive failure rather than feedback, turning normal fluctuation into pathology (Sahib et al., 2024).

Moreover, algorithmic reassurance reframes anxiety as noise instead of information. The reflective question “What is this anxiety trying to tell me?” is replaced by “How do I silence it?” This subtle shift marks a cultural transition from meaning-making to maintenance. Emotional experiences may increasingly be approached through optimization-oriented frameworks rather than reflective interpretation. The self becomes a system to debug rather than a narrative to live.

## Emotional autonomy and the loss of effort

At the motivational level, the paradox intersects with autonomy and competence. Resilience is not built by comfort alone but by effortful regulation—the gradual internalization of coping strategies. Developmental psychology shows that autonomy in emotional control arises from repeated, effortful encounters with challenge. AI comfort technologies interrupt this cycle by providing effortless regulation (Ellikkal and Rajamohan, 2025).

The metaphor of automatic stabilizers in modern aircraft captures the issue well. They keep the plane level but prevent pilots from feeling turbulence, reducing skill development (Prunkl, 2024). Similarly, AI stabilizers keep emotional life level, reducing the micro-failures that teach endurance.

According to self-determination theory, wellbeing rests on three pillars: autonomy, competence, and relatedness. Comfort algorithms mimic relatedness through simulated empathy, yet they undermine autonomy (self-initiation) and competence (self-efficacy) (Catena et al., 2025; Huo et al., 2025). Users feel cared for but not capable. In psychological terms, the regulation becomes controlled rather than autonomous—driven by external cues rather than internal agency. Over time, the user may lose the confidence to face distress without technological mediation (Latikka et al., 2023).

This erosion of autonomy has cultural consequences. A society accustomed to immediate digital comfort may redefine resilience as the ability to stay calm rather than the ability to recover meaningfully. The first seeks suppression; the second seeks transformation. From a theoretical perspective, this argument contributes to autonomy-based models of wellbeing by illustrating how AI-assisted emotional regulation may undermine the development of self-efficacy and effortful coping. The section advances existing human–AI interaction theories by positioning emotional effort—not efficiency—as a central requirement for long-term resilience.

The psychological impact of AI-mediated reassurance is unlikely to be uniform across populations. Developmental stage may moderate both vulnerability and resilience within the comfort paradox framework. Adolescents, whose regulatory capacities and identity structures are still forming, may be particularly sensitive to patterns of externalized soothing, as repeated reassurance could shape emerging coping schemas (Oldehinkel and Ormel, 2023; Valkenburg et al., 2022). In contrast, midlife adults using AI tools for situational stress management may integrate such technologies without significant displacement of internal regulation. Older adults seeking companionship through conversational systems may experience benefits linked to social buffering rather than avoidance reinforcement. Recognizing these developmental differences situates the comfort paradox as a conditional dynamic shaped by age, context, and existing regulatory capacity rather than a universal outcome.

## The neuropsychology of effortless relief

At the neurocognitive level, the framework aligns with reinforcement-learning models. Direct neuroscientific evidence examining AI-mediated reassurance remains limited. The neural considerations discussed here therefore represent theoretical extensions grounded in established research on reinforcement learning, dopaminergic reward processing, and conditioning models (Gershman and Lak, 2025). Findings from these domains provide a plausible conceptual basis for considering how repeated external soothing might shape regulatory engagement over time, while remaining inferential rather than empirically demonstrated in AI-specific contexts (Blanco-Pozo et al., 2024).

The comfort paradox may be conceptually interpreted through reinforcement-learning frameworks and existing theories of reward processing. Research on reinforcement learning suggests that relief from anxiety may involve reward-related processes associated with reinforcement and expectancy. Repeated reassuring interactions may function in ways conceptually similar to reinforcement-learning processes by repeatedly signaling safety and relief. Rapid and low-effort reassurance may potentially strengthen reinforcement-based reassurance-seeking patterns (Langdon and Daw, 2020; Lerner et al., 2021).

Some interaction patterns in AI-mediated reassurance may resemble reinforcement dynamics discussed in studies of variable-ratio reward systems. Repeated intermittent reassurance may contribute to recurring reassurance-seeking behaviors and repeated checking patterns. This pattern may share certain behavioral similarities with reassurance-seeking tendencies observed in anxiety-related conditions (Liebenow et al., 2022).

From a theoretical perspective, repeated reliance on external reassurance could influence engagement in self-directed regulatory processes, although direct neuroscientific evidence in AI-mediated contexts remains limited. Over time, repeated external regulation may reduce opportunities for internally initiated emotional regulation. Habitual reliance on external reassurance may reduce opportunities for developing independent coping and self-regulatory capacities (Sousa et al., 2023).

Effortful engagement in emotional regulation has been associated with adaptive coping and resilience development. Activities involving sustained emotional and cognitive engagement may support adaptive self-regulatory functioning. When emotional regulation becomes predominantly externally mediated, opportunities for effortful self-regulation may decrease. Reduced engagement in effortful regulation may limit opportunities for strengthening adaptive regulatory processes.

## Cultural normalization of perpetual ease

Socioculturally, the paradox extends beyond individuals to collective norms. The implications of the comfort paradox may extend beyond individual emotional regulation to broader sociocultural patterns. Contemporary digital wellness cultures may increasingly prioritize emotional stability and self-optimization. Corporate wellness programs track mindfulness min; educational systems reward composure over curiosity; social media idealizes serenity aesthetics (Valenzuela et al., 2025). Emotional equilibrium is marketed as moral superiority.

Technology magnifies this ideology by making comfort measurable. Heart-rate variability, mood scores, and focus streaks quantify serenity. The implicit message is that inner turbulence is an error state. This conflation of emotional variance with malfunction produces what might be called affective perfectionism—a demand for continuous calm incompatible with human authenticity (Minichiello et al., 2024; Vois and Damian, 2020).

Sociologically, this fosters collective avoidance. Instead of addressing structural causes of anxiety—economic insecurity, social fragmentation, climate dread—societies outsource discomfort management to personalized devices. The burden of adaptation shifts from system to individual: if you feel anxious, your app has a new update. The psychological cost is alienation; the political cost is complacency (Kemp et al., 2020; Valenzuela et al., 2025).

Moreover, as comfort becomes normalized, empathy becomes commodified. The increasing availability of subscription-based emotional AI systems may reshape how care and reassurance are experienced. When every feeling can be soothed for a fee, emotional suffering loses its communal function as a call for solidarity.

Emotional engagement with AI-mediated reassurance is also shaped by sociocultural and gendered norms of emotional socialization. Patterns of emotional disclosure, validation-seeking, and avoidance vary across cultural contexts and gendered expectations. For some individuals, AI systems may lower barriers to articulating distress by providing a non-judgmental interface for expression. For others, particularly in contexts where emotional restraint is culturally reinforced, such systems may amplify rumination or habitual avoidance. Acknowledging these variations situates the comfort paradox within a socially embedded framework, recognizing that its psychological effects are mediated by norms governing emotional expression and regulation.

## Reclaiming discomfort as intelligence

Although comfort-oriented technologies can provide important support, excessive reliance on external reassurance may reduce opportunities for adaptive emotional development. Anxiety, properly engaged, is a teacher—it signals values, vulnerabilities, and the edges of growth. The task of psychological design should therefore be not to eliminate anxiety but to make it intelligible.

Four design principles follow:

**1 Transparency over illusion** – Emotional AI must always reveal its artificial character. Being aware of one's interaction with software maintains a reflective distance and disallows emotional over-identification.

**2 Reflective friction** – Rather than providing immediate soothing, the systems could come up with tiny delays or prompts for reflection: “What might this feeling be linked to?” Friction fosters agency.

**3 Graduated autonomy** – Similar to physical therapy, which slowly diminishes external support, emotional tools must gradually reduce their assistance according to user's increase in confidence.

**4 Human re-embedding** – Digital comfort should turn users toward real human interaction. Machines might simulate empathy, but only human beings can burden it with the moral aspect.

These principles shift design from comfort provision to growth facilitation. They align technology with psychology's deeper mission: to enable flourishing through understanding, not avoidance.

## The future of emotional resilience

The development of mental health technologies should prioritize balanced emotional engagement rather than the pursuit of perfect emotional regulation. The most ethical AI companions are those that at times, consciously choose not to comfort by remaining silent or by delaying, or even challenging the users since this may be development's best service. Human therapists already practice this form of restraint, and compassionate technologies should also incorporate similar principles.

In this context, resilience may be better understood as emerging through interactions between individuals, technologies, and social environments. It is the outcome of the dynamic human-system interaction where the human side is now more vulnerable than ever and where there are certain systems in place that not only provide the necessary support but also allow the existence of emotional and spiritual experience. In designing technologies that support resilience, opportunities for uncertainty, reflection, and honest feedback should be preserved. This perspective also recognizes that manageable anxiety may contribute to problem-solving, empathy, reflection, and creativity.

The comfort paradox framework suggests that emotional effort and efficiency should both be considered when evaluating the psychological impact of emotional AI technologies. In the future, the evaluation of emotional AI may depend not only on how effectively it reduces distress, but also on how meaningfully it supports adaptive emotional engagement.

## Conclusion

Artificial intelligence today is the mediator between human thoughts and feelings. Although AI is often presented as a solution for anxiety, repeated reliance on AI-mediated reassurance may gradually reduce engagement in independent coping processes when relief becomes effortless. The comfort paradox describes this irony: although AI-mediated reassurance may reduce immediate distress, repeated reliance on such systems may inadvertently reinforce avoidance-based coping patterns.

Emotional resilience may involve not only maintaining stability during distress, but also engaging uncertainty with reflection and adaptability. A major challenge for future psychological and technological research is balancing digital emotional support with opportunities for adaptive coping and resilience development. Emotional stability often develops through repeated engagement with manageable distress rather than through its continuous elimination; collective resilience may depend on the capacity to tolerate uncertainty rather than on its systematic avoidance. Emotional intelligence may be better understood not as the maintenance of uninterrupted calm, but as the capacity to tolerate, interpret, and learn from discomfort.

Future research should empirically examine the comfort paradox through longitudinal and experimental designs. Testable questions include whether high-frequency use of AI-mediated reassurance predicts reduced tolerance for uncertainty over time, whether immediate vs delayed algorithmic soothing differentially affects self-efficacy in emotional regulation, and whether reflective prompts attenuate reinforcement-based dependence. Comparative studies across developmental stages could clarify whether adolescents exhibit greater susceptibility to externalized regulation than adults. Neurocognitive investigations may further explore whether habitual AI reassurance alters engagement of prefrontal regulatory networks relative to effortful self-regulation. By operationalizing frequency, design features, and user characteristics, future work can determine when AI-mediated comfort supports resilience and when it risks undermining it.
